# Efficient differentiation of cardiomyocytes and generation of calcium-sensor reporter lines from nonhuman primate iPSCs

**DOI:** 10.1038/s41598-018-24074-y

**Published:** 2018-04-12

**Authors:** Yongshun Lin, Huimin Liu, Michael Klein, John Ostrominski, So Gun Hong, Ravi Chandra Yada, Guibin Chen, Keron Navarengom, Robin Schwartzbeck, Hong San, Zu-Xi Yu, Chengyu Liu, Kaari Linask, Jeanette Beers, Lugui Qiu, Cynthia E. Dunbar, Manfred Boehm, Jizhong Zou

**Affiliations:** 1 iPSC Core, National Heart, Lung, and Blood Institute (NHLBI), National Institutes of Health (NIH), Bethesda, Maryland USA; 2 Laboratory of Cardiovascular Regenerative Medicine, Center for Molecular Medicine, NHLBI, NIH, Bethesda, Maryland USA; 3 Uniformed Services University of Health Sciences, Bethesda, Maryland USA; 4 Hematology Branch, NHLBI, NIH, Bethesda, Maryland USA; 5 State Key Laboratory of Experimental Hematology, Institute of Hematology and Blood Diseases Hospital, Chinese Academy of Medical Science and Peking Union Medical College, Tianjin, China; 6 Genome Techology Branch, National Human Genome Research Institute, NIH, Bethesda, Maryland USA; 7 Pathology Core, NHLBI, NIH, Bethesda, Maryland USA; 8 Transgenic Core, NHLBI, NIH, Bethesda, Maryland USA

## Abstract

Nonhuman primate (NHP) models are more predictive than rodent models for developing induced pluripotent stem cell (iPSC)-based cell therapy, but robust and reproducible NHP iPSC-cardiomyocyte differentiation protocols are lacking for cardiomyopathies research. We developed a method to differentiate integration-free rhesus macaque iPSCs (RhiPSCs) into cardiomyocytes with >85% purity in 10 days, using fully chemically defined conditions. To enable visualization of intracellular calcium flux in beating cardiomyocytes, we used CRISPR/Cas9 to stably knock-in genetically encoded calcium indicators at the rhesus AAVS1 safe harbor locus. Rhesus cardiomyocytes derived by our stepwise differentiation method express signature cardiac markers and show normal electrochemical coupling. They are responsive to cardiorelevant drugs and can be successfully engrafted in a mouse myocardial infarction model. Our approach provides a powerful tool for generation of NHP iPSC-derived cardiomyocytes amenable to utilization in basic research and preclinical studies, including *in vivo* tissue regeneration models and drug screening.

## Introduction

Given a unique capacity to generate any cell type of the body and an ability to self-renew indefinitely, pluripotent stem cells (PSCs), including embryonic stem cells (ESCs) and induced pluripotent stem cells (iPSCs), offer great promise for clinical applications^1–3^. Cardiomyocytes derived from PSCs are in high demand due to broad applications in drug screening, cardiac cytotoxicity evaluation, and development of regenerative cellular therapy approaches to treat heart disease^4,5^. To generate large numbers of cardiomyocytes for these purposes, multiple serum-containing or chemically defined differentiation conditions for human pluripotetent stem cells (hPSCs) have been reported, including one previously published by our group^6–10^. These robust cardiac differentiation methods have provided cells for cardiac drug testing *in vitro*^4,11^ and xenotransplantation of human PSC-derived cardiomyocytes (hPSC-CMs) in animal myocardial infarction (MI) models, including rodents, pigs, and nonhuman primates (NHPs)^12–14^. Although these animal models provide valuable information regarding some aspects relevant to clinical development of hPSC-CMs, there are numerous limitations of interspecies transplantation between donor cells and recipient animals, including rapid immune rejection or use of highly immunodeficient recipient animals, and lack of physiologic signals allowing full integration, maturation and function of hPSC-CMs.

Because of their physiologic similarities to humans, NHPs are important models for preclinical testing of iPSC-derived cells and translational research^15–17^. Notably, NHP models should permit testing of autologous iPSC-derived cell delivery, with an ability to follow transplanted animals long-term, assessing safety, integration of these cells into target tissues, and impact on tissue function, overcoming some of the limitations of immune-mediated rejection following allogeneic or xenogeneic transplantation, or the severe systemic side-efficts by chronic exposure to immunosuppressive drug regimens post-transplantation^18,19^.

To facilitate autologous transplantation in NHP models, fast, reproducible and efficient differentiation protocols to derive desired cell types from iPSCs are essential. To achieve this goal, we generated iPSC lines from rhesus macaques, developed and optimized a robust protocol to differentiate rhesus iPSCs (RhiPSCs) into cardiomyocytes, achieving >85% purity within 10 days without additional enrichment steps, in the presence of fully chemically-defined conditions. To permit insights into the biology and function of these rhesus iPSC-derived cardiomyocytes (RhiPSC-CMs) in different assays, we used CRISPR/Cas9-mediated targeting of the AAVS1 safe harbor locus to generate stable RhiPSC lines that express various genetically encoded fluorescent calcium indicators (GECIs)^20–23^. Cardiomyocytes differentiated from RhiPSCs expressing GCaMP6s, one of the GECIs^21^, displayed strong visible GFP-like fluorescence transients that reflect calcium flux with contraction. The cell lines and protocol we report here should provide investigators an approach for generation of batches of cardiomyocytes differentiated from rhesus monkey iPSCs for *in vitro* drug testing, electrophysiological assays, as well as *in vivo* autologous or allogeneic transplantation.

## Results

### Generation of integration-free iPSCs from rhesus CD34+ hematopoietic stem/progenitor cells (HSPCs)

**Figure 1:**
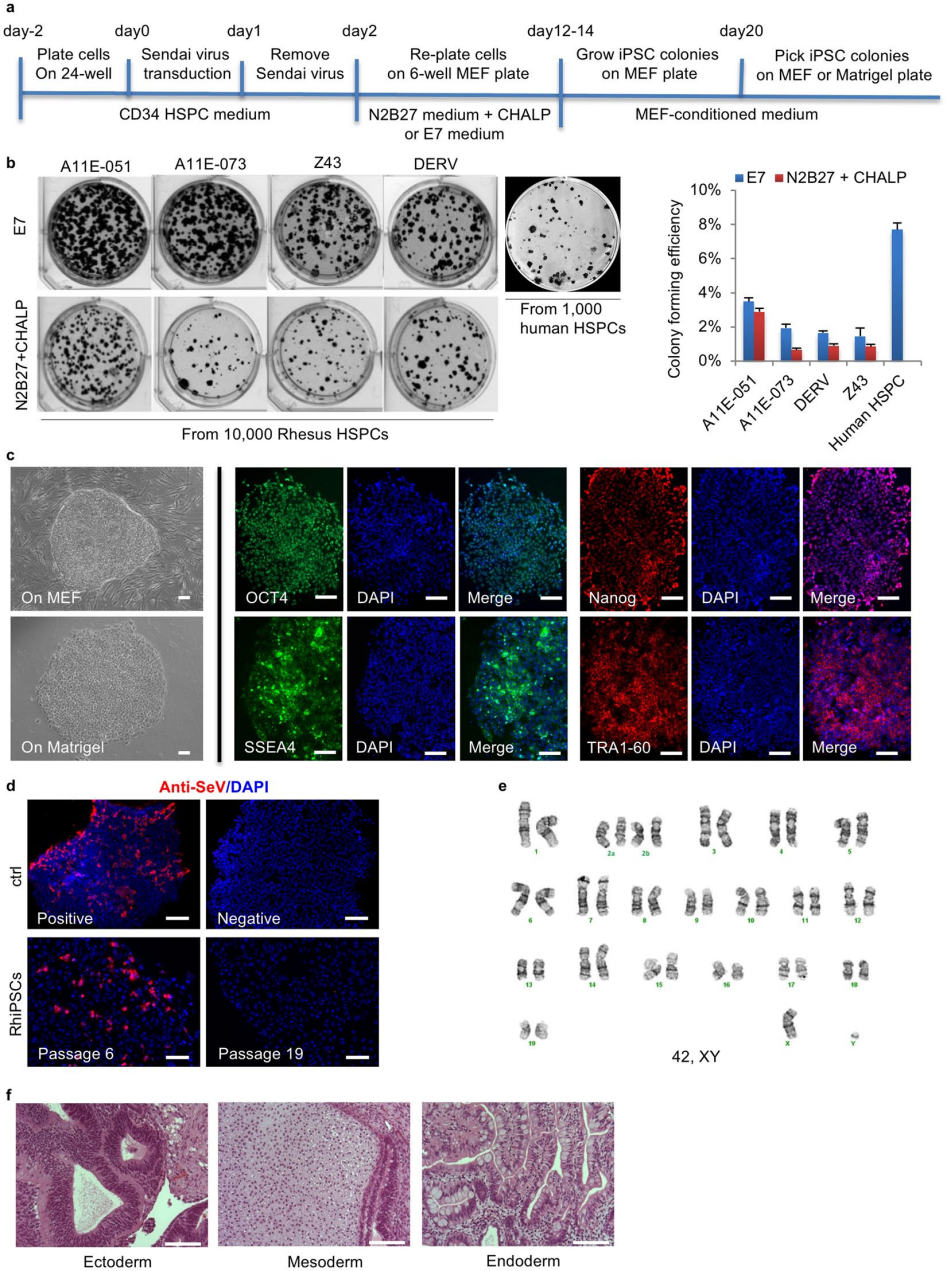
Reprogramming of iPSCs from rhesus CD34+ HSPCs by integration-free Sendai virus. (**a**) Schematic of iPSC reprogramming from rhesus CD34+ HSPCs by Sendai virus. Colonies were formed on MEF and picked onto MEF or Matrigel-coated plates. (**b**) Left panel: Alkaline Phosphotase (ALP) staining of reprogrammed RhiPSC colonies from CD34+ HSPCs of four different rhesus monkeys under E7 or N2B27+ CHALP culture conditions. Right panel: the colony formation efficiencies (y-axis) calculated as the percentage of ALP+ colonies per 10,000 HSPCs under E7 or N2B27+ CHALP conditions. The efficiency of colony formation from 1000 human CD34+ HSPCs was used as comparison (data are mean ± SEM, n = 3). (**c**) Morphology and pluripotency markers immunofluorescence staining of RhiPSC colonies. Scale bars, 100 µm. (**d**) Immunostaining for anti-Sendai virus antibody. Scale bars, 100 µm. (**e**) Normal karyotype of a representative RhiPSC clone (ZH26-HS16). (**f**) H&E staining of sectioned teratomas formed from rhesus iPSCs. Scale bars, 100 µm.

Cytotune® 2.0 Sendai virus (SeV) vectors encoding hOct3/4, hSox2, hKlf4 and hc-Myc^24^ have been widely used to derive transgene integration-free iPSCs from human somatic cells. Previouly we reported on the derivation of one RhiPS line via Sendai reprogramming^25^. In the current study, we compared reprogramming efficiency between two culture conditions. After SeV transduction of CD34+ cells (MOI = 10), iPSC-like colonies appeared by day 6 and were then cultured for 12–14 days on a MEF (mouse embryonic fibroblast) feeder layer in either N2B27 medium plus a combination of small molecules CHALP^26^ or E8 medium without TGF-beta (E7)^27^, before switching to MEF conditioned media (MEF-CM). RhiPSC clones were picked by day 20 and maintained in MEF-CM on MEF or Matrigel (Fig. 1a). The RhiPSC reprogramming procedure and timeline are similar to those for human iPSC (hiPSC) reprogramming. The E7 media resulted in higher reprogramming efficiency (1.5–3.5%) for rhesus CD34+ cells than the N2B27-based media (0.7–2.9%), but still a lower efficiency than human CD34+ cell reprogramming (7.7%), judged by Alkaline Phosphotase (ALP) staining for iPSC clones at day 20 (Fig. 1b).

When cultured in MEF-CM on Matrigel with mechanical passaging, the RhiPSCs exhibited a flat morphology with sharp edges and a high nucleus-to-cytoplasm ratio, similar to hiPSCs. However, the RhiPSC colonies were less compact than hiPSC colonies on Matrigel (Fig. 1c left panels) and could not be maintained in terms of colony morphology and expression of pluripotency markers when passaged using EDTA or enzymatic dissociation, both well-tolerated for passaging of hiPSCs. Immunofluorescence staining for OCT4, TRA-1-60, NANOG, and SSEA-4 confirmed the expression of canonical pluripotency markers in these established RhiPSCs (Fig. 1c right panels). As a single-stranded RNA virus that only replicates in the cytoplasm and does not integrate into the cellular genome, SeV can be eliminated from the cells with passaging^28^. Immunofluorescence staining with an anti-SeV antibody was used to confirm the loss of SeV in the established rhesus iPSCs. The results showed that by passage 19, Sendai virus was undetectable (Fig. 1d). Karyotyping confirmed a normal karyotype of these RhiPSC lines (Fig. 1e). To further confirm pluripotency, the RhiPSCs were injected subcutaneously into NSG immunodeficient mice. Teratomas containing three germ layers (ectoderm, mesoderm, and endoderm) were observed by 6 weeks after injection (Fig. 1f). Using this optimized SeV reprogramming method, we have generated multiple iPSC lines from 6 rhesus monkeys, and all the iPSC lines can be maintained in pluripotent state for at least 40 passages under the culture condition.

**Figure 2:**
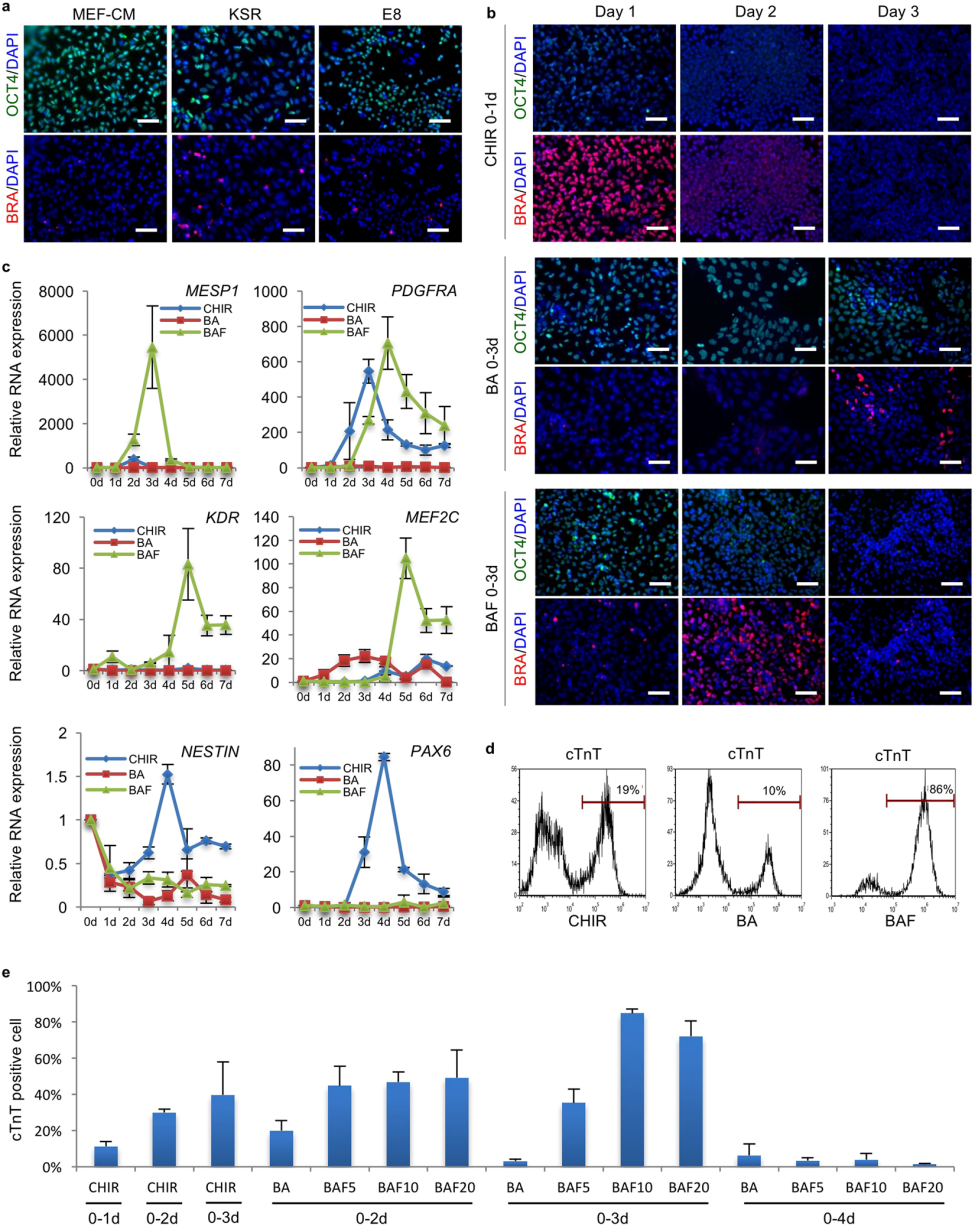
Optimization of mesoderm induction for cardiac differentiation from RhiPSCs. (**a**) Immunofluorescence staining for pluripotency marker OCT4 and early mesoderm marker Brachyury (BRA) on RhiPSC cultured in different media after EDTA dissociation. Nuclei were stained by DAPI. Scale bars, 100 µm. (**b**) Immunofluorescence staining for OCT4 and Brachyury (BRA) during the first three days’ differentiation to access the dynamic profiles of losing pluripotency and gaining mesoderm identities under different mesoderm induction treatments. Nuclei were stained by DAPI. Scale bars, 100 µm. (**c**) Real-time RT-PCR showed daily expression profiles of cardiac progenitor markers (MESP1, PDGFRα, KDR, and MEF2c) and neural progenitor markers (NESTIN and PAX6) under different differentiation conditions (data are mean ± SEM, n = 3). (**d**) Representative flow cytometry analysis from more than 5 times experiments of cTnT expression under CHIR, BA or BAF treatment as shown in (B). (**e**) Screening of different mesoderm induction conditions in RhiPSC cardiac differentiation. Flow cytometry results of cTnT antibody at 10 days after differentiation were shown. The number after letter F was the concentration (ng/ml) of FGF2. CM, conditioned medium; KSR, knockout serum replacement; CHIR, CHIR99021; BA, BMP4 + Activin A; BAF, BMP4 + Activin A + FGF2.

Since the RhiPSCs were maintained using mechanical passaging, we used EDTA to dissociate the colonies into single cells or small clumps before attempted differentiation. We first compared different culture media for ability to maintain the pluripotency of EDTA-dissociated cells prior to differentiation. Immunofluorescence of pluripotency marker OCT4 and the early mesodermal differentiation marker Brachyury suggested that MEF-CM best maintained pluripotency of the RhiPSCs after EDTA dissociation, as the fewest Brachyury positive mesodermal cells were observed in the MEF-CM cultured group. Both KSR (Knockout Serum Supplement) and E8 media caused loss of OCT4 expression and gain of Brachyury expression (Fig. 2a). Per these results, we employed EDTA to dissociate the RhiPSC colonies, and pre-cultured them in MEF conditioned media before inducing differentiation.

### Robust cardiac differentiation from RhiPSCs in fully chemically defined conditions

We previously developed an efficient protocol to differentiate hPSCs into cardiomyocytes by applying 5 uM GSK3β inhibitor CHIR99021 (CHIR) and 3uM Wnt inhibitor IWP2 to sequentially modulate Wnt signaling in a fully chemically defined cardiac differentiation basal medium (CDBM))^10^. To test whether the human differentiation protocol could be applied to RhiPSCs, we initially performed differentiation following our published protocol for hPSCs. However, differentiation to contracting cardiomyocytes was very rare, and instead we frequently observed cells with neural morphology, particularly when extending the differentiation cultures beyond two weeks. Immunofluorescence staining of the differentiated cells for a cardiomyocyte marker (cardiac troponin T, cTnT) and a neuronal marker (Tuj1) revealed a mixture of cells including both cardiomyocytes and neurons (Supplementary Fig. S1a). We attempted to optimize the protocol for rhesus cells by comparing various durations of CHIR and IWP2 treatments. Prolonging exposure to CHIR from 1 day to 2–3 days increased the cardiomyocyte derivation efficiency, as assessed by cTnT staining, but never to greater than 50% (Supplementary Fig. S1b).

We next assessed whether early mesodermal differentiation was occurring from the RhiPSCs, via following OCT4 and Brachyury expression by immunofluorescence staining of the cells over time during the first 3 days after beginning differentiation. Other than the chemical CHIR, the growth factors BMP4, Activin A, and FGF2 have been used to initiate and support mesoderm induction from mouse and human PSCs^6^. Therefore, we tested the modulation of OCT4 and Brachyury expression after treatment with different combinations of these growth factors. We found that CHIR treatment of RhiPSCs reduced OCT4 expression while robustly inducing Brachyury expression at day 1 (Fig. 2b), suggesting there was efficient induction of early mesoderm driven by CHIR treatment, despite eventual low efficiency of cardiomyocyte differentiation. After treating the RhiPSCs with combined growth factors for three days, Brachyury was only turned on in a small fraction of the cells at day 3 with the combination of BMP4 (10 ng/ml) + Activin A (10 ng/ml) (BA), but turned on in most of the cells at day 2 with a combination of BMP4 (10 ng/ml) + Activin A (10 ng/ml) + FGF2 (10 ng/ml) (BAF). However, the staining intensity for Brachyury was lower than that at day 1 with CHIR treatment (Fig. 2b). The temporal dynamics of OCT4 and Brachyury expression suggested different pathways to mesoderm induction by CHIR, BA, and BAF treatments.

We went on to compare the modulation of mesodermal and early cardiac progenitor-associated RNA transcripts under these three different culture conditions. As shown in Fig. 2c, BAF induced significant expression of MESP1, KDR, and MEF2C, comparing with a very low level induction of these genes by CHIR during differentiation. While PDGFRα expression was induced by both CHIR and BAF at comparable levels, the time to peak expression differed by one day. Consistent with our observations of cell morphology and immunostaining results (Supplementary Fig. S1a), we found that expression of the neural progenitor cell marker NESTIN and PAX6 was up-regulated at day 4 in the CHIR treatment condition, demonstrating that CHIR alone was insufficient to drive RhiPSCs to cardiac mesoderm, and instead supported differentiation towards neural ectoderm. Representative cardiac differentiation results for all three mesodermal induction conditions (5 uM CHIR for 1 day, 10 ng/ml BA for 3 days, or 10 ng/ml BAF for 3 days) are shown in Fig. 2d, demonstrating that BAF treatment could induce cardiac differentiation to a level of over 85% purity. Next, we investigated the optimal timing of exposure to the cytokines BMP4 (10 ng/ml) and Activin A (10 ng/ml), and the concentration of FGF2 for cardiomyocyte generation. Exposure of RhiPSCs to 3 days of BMP4 (10 ng/ml), Activin A (10 ng/ml) and FGF2 (10 ng/ml) produced cardiomyocytes at the highest purity, reproducibly greater than 85%, without requiring cell sorting or metabolic selection (Fig. 2e and Supplementary Video S1)^29^.

To better understand the role of FGF2 in the early cardiac mesoderm induction, we compared expression of pluripotency and early mesodermal genes during the first four days in culture treatment with different concentrations of FGF2 (from 0 to 20 ng/ml) in combination with fixed concentrations of BMP4 and Activin A. As shown in Supplementary Fig. S2, the expression of pluripotency markers NANOG and POU5F1 peaked at day 1 after BA or BAF treatment. However, although POU5F1 expression maintained at high levels at day 2, the addition of FGF2, especially at 10 and 20 mg/ml, significantly reduced its expression at day 3 relative to BA treatment alone, consistent with our immunofluorescence staining results. The addition of FGF2 resulted in high Brachyury expression at day 2. MESP1, the earliest cardiac mesoderm marker, became detectable at day 2 and peaked at day 3. The comparison of cell morphology changes under different treatments is shown in Supplementary Fig. S3. The coincident expression of MESP1 with POU5F1 and T during RhiPSC-cardiomyocyte differentiation is reminiscent of mouse ESC studies showing Mesp1 is a direct target of Oct4 and Brachyury^30,31^. FGF2 also significantly induced expression of PDGFRa, an early cardiovascular progenitor marker, and AXIN2, the Wnt/β-catenin signaling target. The expression of both PDGFRa and AXIN2 continued to increase at day 4 even after withdrawal of BAF and addition of the Wnt inhibitor IWP2 at day 3. These results suggest that FGF2 plays an important role in the dynamic change from self-renewal to meseodermal differentiation through modulating canonical Wnt signaling, the precise balance of which determines selective induction of cardiac mesoderm in RhiPSCs.

**Figure 3:**
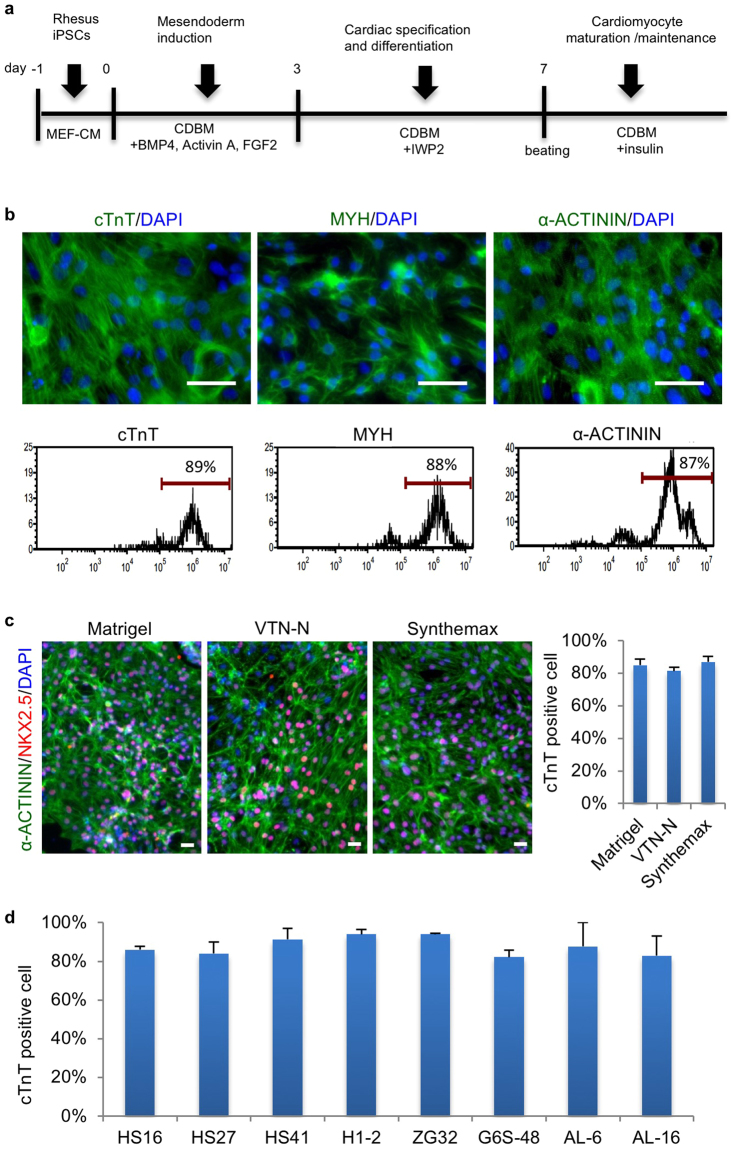
Robust cardiomyocyte differentiation from RhiPSCs in fully chemically defined conditions. (**a**) Schematic of cardiomyocyte differentiation protocol from RhiPSCs. The differentiation stages and media were shown above and under the arrows and timeline, respectively. MEF-CM, MEF conditioned medium; CDBM, cardiac differentiation basal medium. (**b**) Immunofluorescence staining (top panels) and FACS analysis (bottom panels) for cardiac markers: cTnT, MYH, and α-ACTININ at day 10 of differentiation. Nuclei were stained by DAPI (blue). Scale bars, 50 µm. (**c**) Cardiac differentiation efficiency on different matrices (Matrigel, VTN-N, and Synthemax). Representative immunofluorescence staining for α-ACTININ and NKX2.5 (left panels) and flow cytometry analysis for cTnT (right panel) (data are mean ± SEM, n = 3). Nuclei were stained by DAPI. Scale bars, 50 µm. (**d**) Cardiac differentiation efficiency, assessed by cTnT expression via flow cytometry, of 8 unique RhiPSC lines on Synthemax-coated plates (data are mean ± SEM, n = 3).

Based on these optimization steps, we have now developed a highly efficient protocol to robustly induce beating cardiomyocyte differentiation from RhiPSCs in 7–10 days (Fig. 3a). From RhiPSCs seeded at the density of 3 × 10^4^ cells/cm^2^, we routinely derived cardiomyocytes at the density of 1–2 × 10^5^ cells/cm^2^ after 10 days of differentiation, representing a 5-fold increase of cell number. Taken together, we are able to generate 5–10 × 10^6^ cardiomyocytes per 6-well plate, providing the capacity to produce large numbers (10^8^–10^9^) of cardiomyocytes for *in vivo* transplantation. The RhiPSC-CMs were further shown via immunofluorescence staining and FACS to express multiple cardiac muscle markers (NKX2.5, cTnT, MYH (myosin heavy chain) and α-ACTININ) with >85% purity (Fig. 3b,c).

Matrigel was the extracellular matrix material initially used to develop the protocols for iPSC maintenance and cardiac differentiation in this study, based on widespread use for human iPSC differentiation. However, Matrigel is a poorly defined product of a murine transformed cell line, and iPSC-derived cells grown on Matrigel were previously shown to be rejected in an autologous rhesus teratoma model^15,32^. We sought a fully xeno-free and chemically defined differentiation protocol, and tested growth on defined matrix surfaces including recombinant human vitronectin (VTN-N) and Synthemax^33^. RhiPSCs could be maintained on all of three matrices with comparable morphology in MEF conditioned media under mechanical passage (Supplementary Fig. S4a). The differentiation efficiency was comparable using the same RhiPSC line grown on Matrigel versus VTN-N or Synthemax coated plates, all over 80% TnT positive after 10 days of culture (Fig. 3c,d and Supplementary Fig. S4b). These results were confirmed in 8 RhiPSC lines from 4 different rhesus monkeys.

### Further Characterization of RhiPSC-CMs

**Figure 4:**
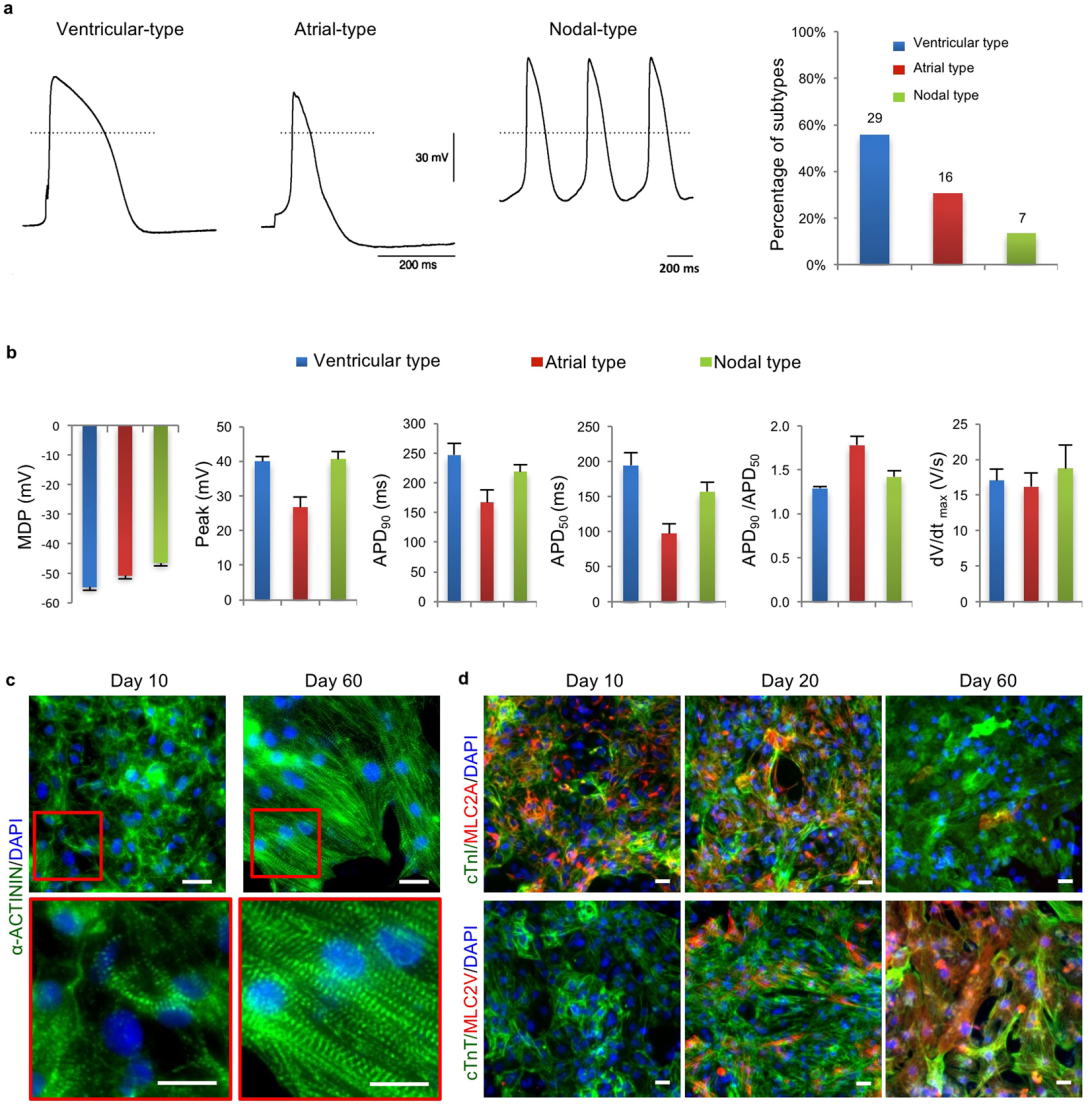
Further characterization of RhiPSC-CMs. (**a**) Representative action potentials from patch clamp recordings of the three cardiomyocyte substypes (ventricular, n = 29; atrial, n = 16; and nodal, n = 7) at day 20 of differentiation. (**b**) Patch clamp recording of MDP, peak voltage, APD at different levels of repolarization (90% and 50%), and dV/dt_max_ rate from three cardiomyocyte subtypes (ventricular, n = 26; atrial, n = 19; and nodal, n = 7) at day 15–20 of differentiation. (**c**) Representative immunofluorescence staining for α-ACTININ from more than 3 experiments showed increased sarcomere alignment in RhiPSC-CMs from 10 days to 60 days of culture. The details within red rectangle boxes in upper panels are shown in the lower panels. Nuclei were stained by DAPI. Scale bars, 25 µm. (**d**) Co-staining for MLC2A and MLC2V with cTnI or cTnT showed that most cells expressed MLC2A at early stage (10 days) but expressed MLC2V at late stage (60 days), while a mix population of MLC2A and MLC2V expressing cells was observed at day 20. Nuclei were stained by DAPI. Scale bars, 25 µm.

We applied whole-cell patch-clamping to analyze electrophysiological characteristics of RhiPSC-CMs 20 days after induction of differentiation. As shown in Fig. 4a, we observed typical action potential shapes characteristic of all three cardiomyocyte subtypes: 29 (56%) ventricular-like cells, 16 (31%) atrial-like cells, and 7 (13%) nodal-like cells from 52 analyzed cardiomyocytes. Ventricular-like cardiomyocytes showed a long plateau phase, atrial-like cardiomyocytes showed a high ratio of APD_90_/APD_50_ (action potential duration at 90% vs 50% levels of repolarization), and nodal-like cardiomyocytes showed automaticity and a prominent phase 4 depolarization. Further analysis showed that day 20 RhiPSC-CMs have MDP (maximal diastolic potential) of −55 mV~−47 mV and dV/dt_max_ (maximal rate of depolarization) of 16~19 v/s (Fig. 4b), which is comparable to the reported parameters for human iPSC-derived immature cardiomyocytes^8,34^.

To investigate the maturation potential of the RhiPSC-CMs, we continued to culture the cells for 2 months and compared them with the cells at 10 days of differentiation. Immunofluorescence staining of α-ACTININ showed a significant increase in sarcomere alignment at day 60 when compared with day 10 (Fig. 4c), suggesting increased maturation by day 60^35^. The expression patterns of the two major isoforms of cardiac Myosin Light Chain 2 (MLC2A and MLC2V) have also been used to asesss maturation of cardiomyocytes^36^. In developing hearts or *in vitro* at early maturation stages, MLC2A is expressed in the majority of both atrial and ventricular cardiomyocytes. In adult hearts or after long-term *in vitro* maturation, the ventricular cardiomyocytes lose MLC2A expression but gain expression of MLC2V^8,10^. Co-staining for cardiac troponins with MLC2A or MLC2V in our RhiPSC-CMs at 10, 20, and 60 days are shown in Fig. 4d. At day 10, all the cardiomyocytes (cTnI (cardiac troponin I) or cTnT positive) expressed MLC2A but not MLC2V. At day 20, although most of the cardiomyocytes retained some degree of MLC2A expression, expression of MLC2V was detectable in many cells, indicating maturation. By day 60, the majority of cardiomyocytes expressed MLC2V but not MLC2A, consistent with a mature ventricular phenotype. All these results demonstrate that our RhiPSC-CMs have the capacity to mature with long-term culture.

### *In vivo* transplantation of RhiPSC-CMs into NSG mice

**Figure 5:**
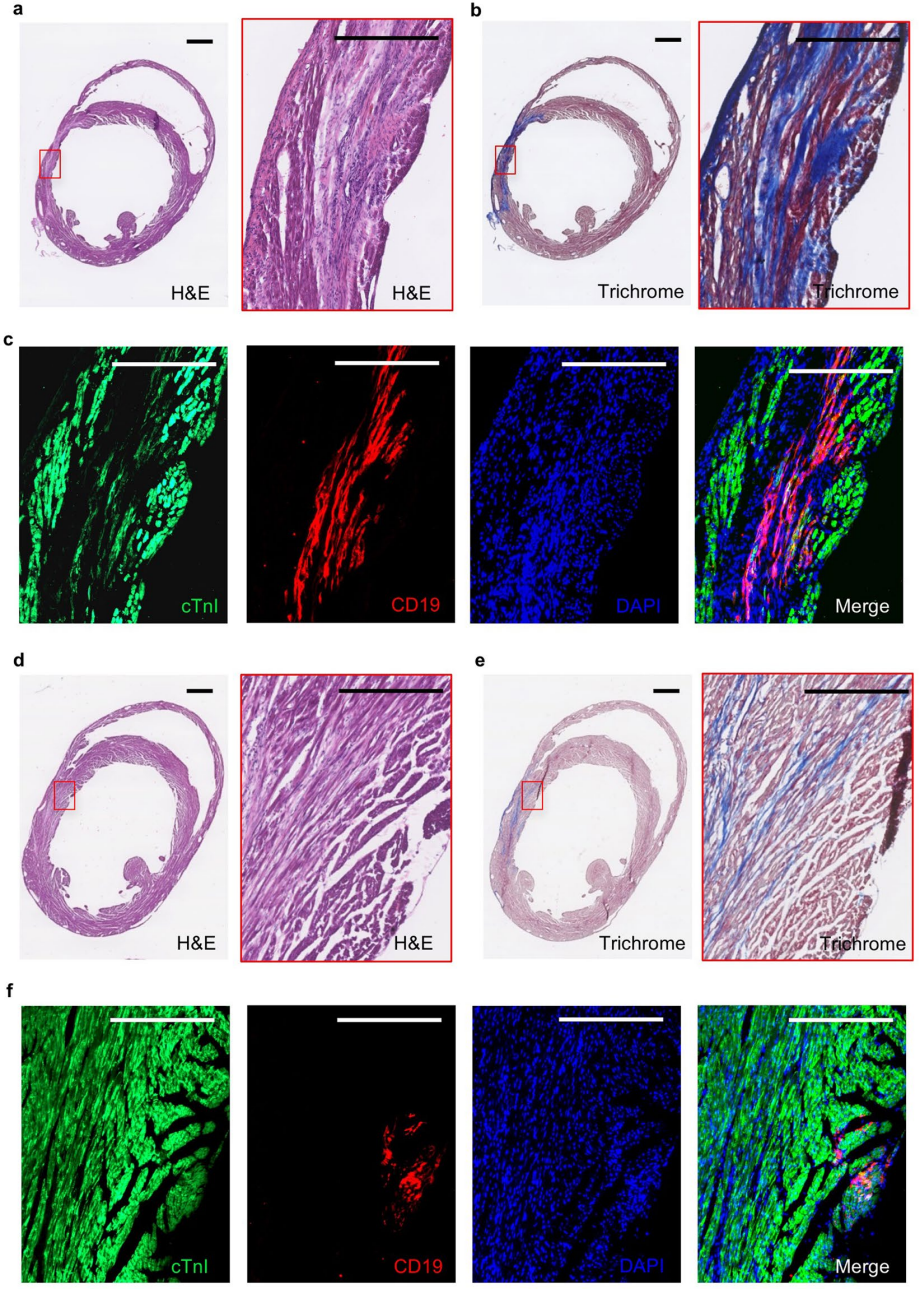
Engraftment of hΔCD19-labeled RhiPSC-CMs into NSG mouse myocardial infarct area. (**a**–**c**) Incorporation of hΔCD19-labeled RhiPSC-CMs in the central myocardial infarct area. (**a**) H&E staining of the myocardial infarct at two months after LAD ligation and RhiPSC-CM transplantation. (**b**) Masson’s trichrome staining to visualize the infarcted cardiac tissue (blue color indicates fibrotic tissue). (**c**) Immunofluorescence staining of CD19 positive cells located throughout infarct area, these cells were also positive for cTnI co-staining. Scale bars, 500 µm. (**d**–**f**) Distribution of hΔCD19-labeled RhiPSC-CMs in the peri-infarct area. (**d**) H&E staining of the infarct area of heart tissue at two months after LAD ligation. (**e**) Masson’s trichrome staining to visualize the infarct area. (**f**) Immunofluorescence staining revealed CD19 (red) positive cells were outside the infarct area but positive with cTnI co-staining. Scale bars, 400 µm.

We assessed the *in vivo* engraftment ability of RhiPSC-CMs in immune-deficient NSG mice, using cardiomyocytes generated from RhiPSC lines expressing a non-immunogenic human truncated CD19 (hΔCD19) marker gene via CRISPR/Cas9 gene knock-in at the rhesus AAVS1 safe harbor locus^25^. CD19 is a B cell-restricted marker, hence is not expressed in other cell types in the body^37^. Day 12 beating cardiomyocytes were derived from hΔCD19-labeled RhiPSCs and injected into the myocardium of immune-deficient NSG mice after ligation of the left anterior descending (LAD) artery without reperfusion. Two months after transplantation, the mice were sacrificed and frozen cross-sections of the hearts were stained by H&E and Masson’s trichrome^38^ to identify the infarct areas (Fig. 5). The antibodies for cTnI and CD19 were applied to locate injected RhiPSC-CMs. CD19-positive cells could be found in both the central- (Fig. 5a–c) and peri-infarct (Fig. 5d–f) regions, and the CD19-fluorescence signaling overlapped with cTnI staining, although the intensity of cTnI staining in the infarction area was weaker than in the healthy area, suggesting incomplete maturation of engrafted RhiPSC-CMs.

### Genetically encoded calcium indicators in RhiPSC-CMs

**Figure 6:**
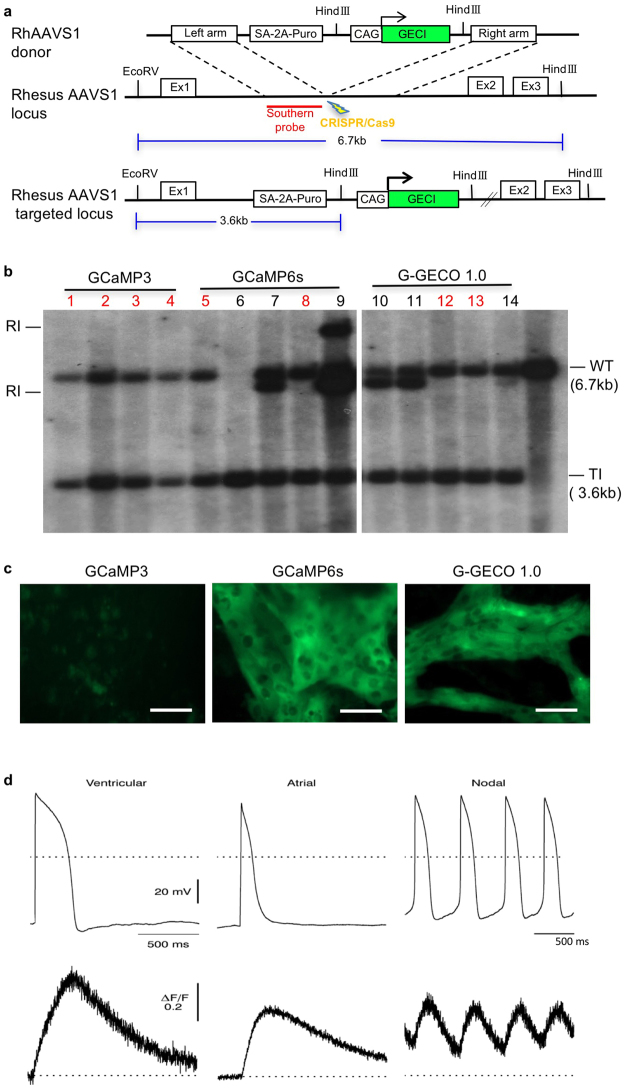
Genetically encoded calcium indicator (GECI) in RhiPSCs for detection of calcium flux in contracting cardiomyocytes. (**a**) Schematic illustration of the AAVS1 gene targeting with GECI mediated by CRISPR/Cas9 in RhiPSCs. Ex, exon. GECI was GCaMP3, GCaMP6s, G-GECO1.0., or R-GECO1.0 (**b**) Southern blot analysis showed the targeted and random integrations in the clones picked from three different green-fluorescence GECI RhiPSC lines. The images shown were cropped from the same full-length Southern blot with all four different GECI lines (Supplementary Fig. S5). Red numbers labeled the clones without random integration. RI, random integration; TI, targeted integration (=3.6 kb); WT, wild type (=6.7 kb). (**c**) Comparison of GFP-like fluorescence intensity of contracting cardiomyocytes derived from three different green-fluorescence GECI RhiPSC lines when the culture medium was changed to Tyrode’s salt solution. Scale bars, 100 µm. (**d**) Coupling of action potential change and calcium flux shown via simultaneous recording by patch clamp and fluorescence imaging of three different cardiomyocyte subtypes derived from GCaMP6s RhiPSCs. Scale bars, 50 µm.

Fluorescent calcium indicators respond to changes of calcium ion concentrations in beating cardiomyocytes, and have been used to visualize the intracellular calcium flux during contraction^22^. To facilitate *in vitro* and *in vivo* applications of these RhiPSC-CMs, we applied CRISPR/Cas9 gene editing technology^39^ to knock-in genetically encoded calcium indicators (GECIs) at the AAVS1 safe harbor locus, as we previously reported for the hΔCD19 marker gene^23,25^. The rhesus AAVS1 locus is actively expressed in rhesus pluripotent stem cells as well as in differentiated cells, and makes it an ideal target for the introduction of cell markers or other transgenes^25^. Donor plasmids containing one of four calcium indicators, GCaMP3, GCaMP6s, G-GECO1.0, and R-GECO1.0^12,20,21,40,41^ and AAVS1 homology arms were constructed and transfected into RhiPSCs along with AAVS1 gRNA and Cas9 (Fig. 6a)^25^. After puromycin selection, surviving colonies were manually picked and expanded. Southern blot analysis using the probe recognizing the left homology arm demonstrated that 40–100% of picked clones had precise gene-targeting without additional random insertions for each of the four different GECIs (Fig. 6b, Supplementary Fig. S5). Clones with single allele targeted integration only were chosen and expanded for further characterization. These gene-edited RhiPSCs maintained normal karyotypes.

Three different green-fluorescence GECI RhiPSC lines, GCaMP3, GCaMP6s, and G-GECO1.0, were differentiated into beating cardiomyocytes to compare the calcium indicator fluorescent intensity. Previous studies reported that G-GECO1.0 produced approximately double the GFP fluorescence intensity of GCaMP3^20^, and GCaMP6s produced more than a 10-fold greater intensity of GCaMP3^42^. We observed similar results in our GECI RhiPSC-CMs. When the culture medium was changed to clear Tyrode’s salt solution to reduce background fluorescence, signal from GCaMP3 RhiPSC-CMs was visible, though appreciably weaker than signals from GCaMP6s and G-GECO1.0 RhiPSC-CMs during contraction (Fig. 6c). In our routine cardiomyocyte maintenance culture medium that contains phenol red, GCaMP6s RhiPSC-CMs still featured a robust optical signal detectable over the fluorescence background (Supplementary Video S2). As such, the GCaMP6s-RhiPSC cell line was chosen for *in vitro* calcium imaging analysis. We demonstrated that the GFP-like signals from contracting GCaMP6s RhiPSC-CMs reflected calcium transients in all three different cardiomyocyte subtypes (ventricular, atrial, and nodal) and were slightly delayed but well-coupled to action potentials recorded by patch clamp. The minor delay of calcium signal increase after action potential upstroke suggests that the voltage-gated calcium channel on the cell membrane functioned properly to trigger calcium influx into RhiPSC-CMs (Fig. 6d).

### Detection of calcium transients and response to drugs impacting action potentials in GECI RhiPSC-CMs

**Figure 7:**
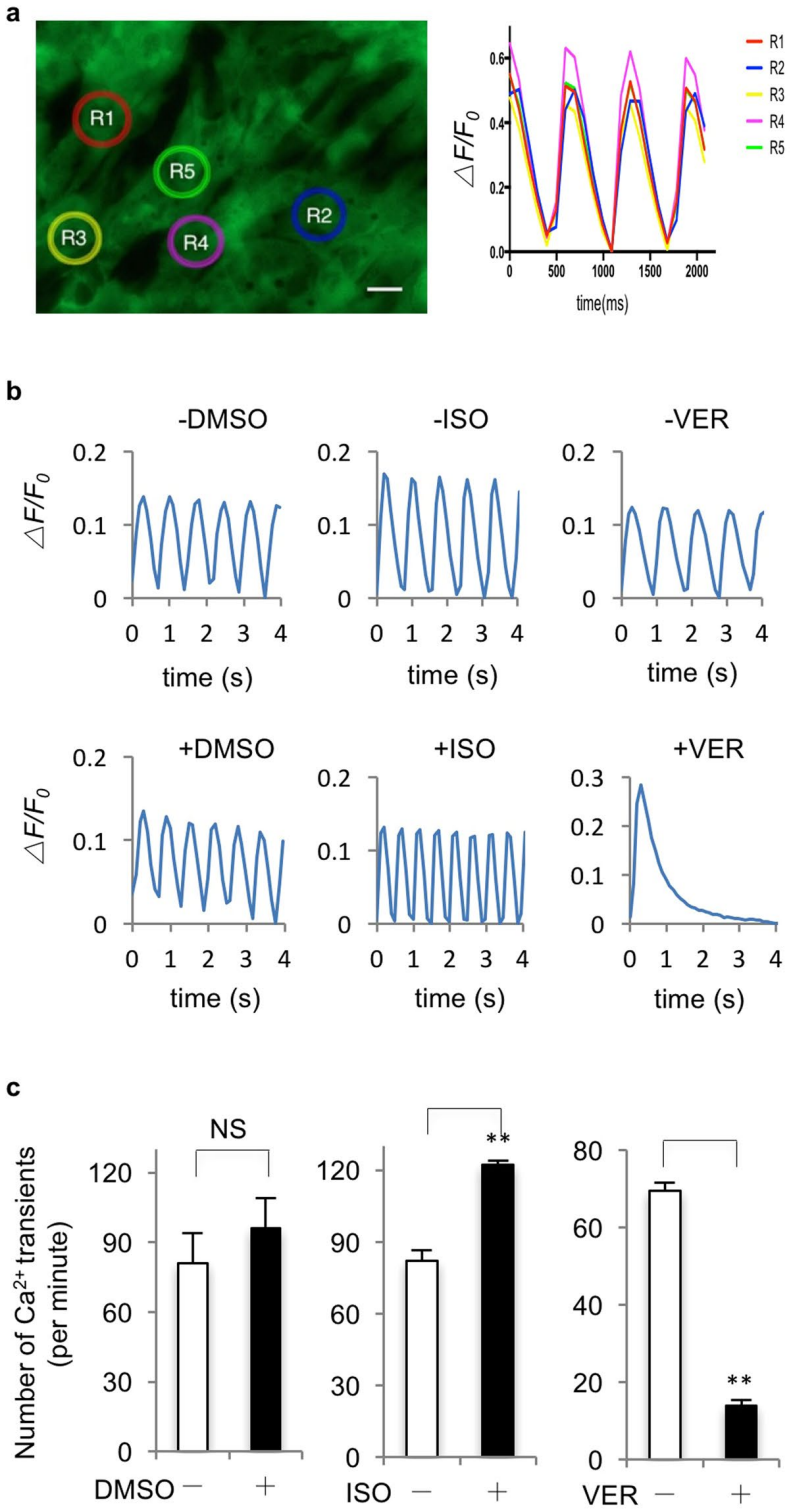
Application of GECI RhiPSC-CMs in calcium flux detection and drug testing. (**a**) Fluorescence recording for calcium flux from 5 randomly selected areas (R1-5) in a field (left panel) showed consistent fluorescence intensity change and slight delay from left to right among different areas of spontaneously beating GCaMP6s RhiPSC-CMs (right panel). (**b**) Representative fluorescence intensity change (*ΔF/F*_0_) from three independent experiments in spontaneously beating GCaMP6s RhiPSC-CMs to assess changes in calcium oscillation frequency between control group (top panels) and drug treated group (bottom panels). ISO, isoproterenol; VER, verapamil. DMSO was used as control. (**c**) Comparison of calcium oscillation frequency (data are mean ± SEM, n = 3) with or without vehicle or drugs. ms, millisecond; s, second; ISO, isoproterenol; VER, verapamil; NS, not significant. **P < 0.01. Scale bars, 25 µm.

To confirm consistent measurement of calcium transients by GECI in beating RhiPSC-CMs, we randomly chose five regions-of-interest (ROI) with same area size on an imaging field (Fig. 7a). The fluorescence recording from GCaMP6s RhiPSC-CMs showed that the changes of fluorescence intensity and frequency were similar among all ROIs, except a slight delay from the rightmost ROI (R2) compared to the others, indicating that connected beating in RhiPSC-CMs was synchronized, and the transmission direction of an action potential that induced calcium flux was from left (R3) to right (R2) in the imaging field. Furthermore, we tested the response of calcium flux in GCaMP6s RhiPSC-CMs after the treatment with drugs that affect cardiomyocyte contraction. Isoproterenol (ISO) is a non-selective β-adrenoreceptor agonist that produces an elevated heart rate^43^. Indeed, a significant increase in the frequency of calcium transients (from 82.1 ± 4.4 times/min to 122.2 ± 1.8 times/min, n = 3, *p* < 0.01) was observed 10 minutes following the treatment with 10uM ISO (Fig. 7b and c, middle panels) by fluorescence imaging when compared to control (0.1% DMSO, from 80.9 ± 13.0 times/min to 95.9 ± 13.2 times/min, n = 3) (Fig. 7b and c, left panels). Conversely, 3 minutes after the treatment with 0.2 uM verapamil (VER), a calcium channel blocker that directly inhibits voltage-dependent calcium channels^44^, calcium oscillation frequency significantly decreased from 69.6 ± 2.0 times/min to 13.9 ± 1.4 times/min (Fig. 7b and c, right panels). Taken together, these results demonstrated that the GECI-expressing rhesus cardiomyocytes could be used to visualize dynamic changes in intracellular calcium concentration, and these cells responded in expected ways to cardioactive drugs.

## Discussion

NHPs are physiologically and developmentally close to humans, hence they represent more appropriate models for preclinical testing of regenerative therapies. The transplantation of hiPSC-CMs into the infarcted myocardium of rhesus monkeys, or allogeneic iPSC-CM transplantation between different cynomolgus monkeys have been recently reported^12,45^. But these xenogeneic and allogeneic studies involved utilization of very intensive immunosuppressive drug regimens to prevent rejection of the transplanted cells, which could interfere with the assessment of host immune response and long-term safety of transplanted iPSC-CMs. Follow-up was necessarily very brief, weeks to a few months at most. As such, autologous transplantation can provide unique insights into long-term safety and efficiency, and serves as a direct preclinical model for personalized human iPSC-based cellular therapies, as demonstrated by a recent study showing that autologous cynomolgus iPSC-derived dopamine neurons provided functional benefits in a Parkinson’s Disease model without using immunosuppression^18^.

Rhesus macaques are the most commonly used NHP for medical research and RhiPSCs have been derived in different labs, including our own^15,46^. Although one report described using embryoid bodies (EB) to differentiate cardiomyocytes from rhesus embryonic stem cells (ESCs) at low efficiency a decade ago^47^ and another recent report described generation of two of the three subtypes of cardiomyocytes (atrial and ventricular but not nodal cardiomyocytes) from RhiPSCs^48^, an efficient method to derive all three types of cardiomyocytes from RhiPSCs is still lacking to date. Here we report an efficient protocol to differentiate RhiPSCs into all three cardiomyocyte subtypes in fully chemically defined conditions, as well as generation of GECI-RhiPSC lines for visualizing calcium flux in cardiomyocytes derived from these RhiPSCs.

Canonical Wnt signaling has been demonstrated to play a biphasic role during *in vivo* heart development and *in vitro* cardiac differentiation in several species^6,49,50^. Because of the advantages of cost and stability, chemical inhibitors have often replaced growth factors to modulate Wnt signaling and efficiently drive cardiac differentiation from human iPSCs. For example, CHIR (GSK3β inhibitor) was used to promote Wnt signaling and then IWP2 (Porcupine inhibitor) was used to block Wnt secretion during two phases of cardiac differentiation^7,8,10^. However, in rhesus iPSC differentiation, treating RhiPSCs with CHIR robustly induced the expression of the early mesoderm marker Brachyury at day 1, but resulted in low efficiency cardiac differentiation with a mixture of neural cells. Instead, we found that the combination of three growth factors BAF (BMP4, Activin A, and FGF2) for mesoderm induction, followed by IWP2 inhibition could drive RhiPSCs to differentiate into highly pure cardiomyocytes in one week. Upon dissection of their effects on the expression of pluripotency and mesoderm genes at the early stages of RhiPSC and hiPSC differentiation (Supplementary Fig. S6), we found that CHIR and BAF induced Brachyury expression at day 1–2 and day 2–3, respectively, when POUF1 was still expressed. MESP1 expression peaked at one day after the peak of Brachyury expression. These suggested co-expression of POUF1 and Brachyury is essential to induce MESP1 expression, and downregulation of Brachyury is associated with upregulation of MESP1. HiPSCs treated with CHIR and RhiPSCs treated with BAF still maintained >50% level of their POUF1 expression when MESP1 expression increased >150-fold, wherase RhiPSCs treated with CHIR only had 10% level of POUF1 expression when MESP1 expression reached 60-fold increase at its peak. This indicates that the level and timeframe of both pluripotency and mesoderm gene expression are critical to direct RhiPSCs into the cardiac cell fate.

Genetically encoded calcium indicators (GECIs) provide an invaluable opportunity to visualize the calcium flux in cardiomyocytes and to better understand individual cell behavior and cell-cell interactions under physiological conditions. In this study, we applied the CRISPR/Cas9 gene editing technique to knock-in GECIs at the rhesus AAVS1 safe harbor locus and compared the fluorescence intensity after the GECI RhiPSC lines were differentiated into beating cardiomyocytes. We selected the GCaMP6s - the indicator with the highest intensity of fluorescence - for further testing and showed that the recording of the GFP-like fluorescence transition in beating GCaMP6S RhiPSC-CMs not only overlapped with the pattern of action potential recording, but also responded properly after drug treatments. These data strongly demonstrate their capacity as an imaging tool for *in vitro* and *ex vivo* cardiac physiological assays and drug screening.

In summary, our fully chemically defined, xeno-free and efficient rhesus iPSC cardiac differentiation method provides an unprecedented opportunity to derive rhesus cardiomyocytes for *in vitro* function tests and *in vivo* cell transplantation, especially enabling the advance of autologous transplantation in a rhesus monkey model that will aid in bringing human cellular therapy to the bedside in the future. Meanwhile, the generation of GECI rhesus iPSC lines enables the visualization of these iPSC-derived cardiomyocytes for better characterization and understanding of their physiological perspectives in different assays. These tools will be invaluable resource for basic research and preclinical testing of cardiac cellular therapy using NHP models.

## Methods

### Animals

All animals used in this study were housed and handled in accordance with protocols approved by the Animal Care and Use Committee of the National Heart, Lung, and Blood Institute.

### Generation of iPSCs from rhesus and human bone marrow CD34+ HSPCs

Rhesus and human bone marrow CD34 + HSPCs were FACS sorted from bone marrow aspirate-derived buffy coat and suspended in HSPC medium (StemSpan SFEM medium (STEMCELL Technologies) containing stem cell factor (SCF, 100 ng/ml), fms-like tyrosine kinase 3 (Flt-3) ligand (100 ng/ml) and human thrombopoietin (TPO, 100 ng/ml)) and cultured in a 24-well plate (1 × 10^5^/well in 1 ml medium) at 37 °C in 5% CO_2_ incubator. The top half of the media was changed every day for two days. At day 0 of reprogramming, 1 × 10^5^ CD34 + HSPCs was placed in one well of a 24-well plate with fresh HSPC medium and transduced with CytoTune-iPS 2.0 Sendai Viruses (Thermo Fisher Scientific) expressing transcription factors hOct3/4, hSox2, hKlf4 and hc-Myc at a multiplicity of infection (MOI) of 5 or 10 for each virus by spinning down the plate at 1200 × *g* for 1 hour. Sendai Viruses were removed at day 1 by centrifuging the transduced cells at 200 × *g* for 10 minutes, and then the cells were resuspended in 1 ml of fresh HSPC medium. At day 2, 10% of rhesus CD34+ cells, which originated from 10,000 day 0 cells before transduction, were placed in one well of 6-well plates pre-seeded with mouse embryonic fibroblasts (MEF) feeders and cultured with either N2B27 medium plus a combination of small molecules (CHIR99021, HA-100, A-83-01, LIF, and PD0325901, known collectively as CHALP) or E8 medium without TGF-beta (E7) for the first 12–14 days. For both conditions, the media were changed to MEF-conditioned medium (MEF-CM) supplemented with 20 ng/ml bFGF until day 20. The media were replaced every other day between day 2 and day 20 and replaced every day after day 20. Colonies with a morphology similar to human ESCs started to appear as early as day 6 after SeV transduction, and were picked manually by day 20 onto MEF or Matrigel-coated plate with MEF-CM and expanded for further characterization and cryopreservation. For human CD34+ cells, only E7 condition was used to compare reprogramming efficiency with monkey CD34+ cells. Both 10% and 1% human CD34+ cells at day 2, which originated from 10,000 and 1,000 day 0 cells respectively, were placed in each well of 6-well plates. Because 10,000/well generated too many human iPSC colonies to count or pick at day 20, only 1,000/well plates were used for Alkaline Phosphotase (ALP) staining and colony counting. While MOI of 5 and 10 were tested, we found no obvious toxicity to CD34+ cells and slightly higher reprogramming efficiency from MOI = 10. Therefore, MOI = 10 conditions were used to compare reprogramming efficiency between human and rhesus CD34+ cells.

### RhiPSCs maintenance, passaging and cryopreservation

RhiPSC lines were routinely maintained in MEF-conditioned medium (MEF-CM) with 20 ng/ml bFGF on Matrigel-coated plates, incubated under hypoxic (5% O_2_) conditions, and manually passaged every 2–4 days. Culture media were replaced every day. The manually picked RhiPSC colonies were cryopreserved in CryoStor CS10 medium (STEMCELL Technologies) and stored in liquid nitrogen. Frozen RhiPSCs were thawed and cultured in MEF-CM with 20 ng/ml bFGF on pre-seeded MEF feeders. The RevitaCell Supplement (Invitrogen) was added in medium for first day of culture after thawing but removed one day later. Colonies were manually passaged and placed on Matrigel-coated plates for routine culturing.

### Cardiomyocyte differentiation from RhiPSCs

The RhiPSCs grown in MEF-CM were dissociated by 0.5 mM EDTA to single cells or small clumps and seeded on Matrigel (or Synthemax or VTN-N) coated plates in MEF-CM with a density of around 30000 cells/cm^2^. Differentiation was started (defined as day 0) when the cells reached aournd 50% confluent (next day) by changing the media from MEF-CM to mesoderm induction media containing cardiac differentiation basal media (CDBM) and combined growth factors (Activin A + BMP4 + FGF2). CDBM consists of E8 basal medium (DMEM/F-12, L-ascorbic acid, sodium selenite, and Holo-transferrin)^27^, 1 × Chemically Defined Lipid Concentrate (100 × , Life Technologies, 11905-031), and 1 × Penicillin-Streptomycin (Life Technologies, 15140-122). The media were changed daily for three days (days 0–3). From day 3 to day 7, the media were changed every other day to cardiac differentiation media containing CDBM and IWP2. At day 7, beating cardiomyocytes were observed and the media was changed to cardiac maintenance media containing CDBM and insulin. The cardiomyocytes can be cultured in CDBM with insulin for long-term maintenance and maturation. The growth factors and inhibitors used in culture were: Activin A (10 ng/ml, R&D, 338-AC/CF), BMP4 (10 ng/ml, R&D, 314-BP/CF), FGF2 (10 ng/ml, Peprotech, 100-18B), Insulin (20 ug/ml, Sigma, I9278), and IWP2 (3 uM, Tocris, 3533).

### Electrophysiological and photometric assay

Cardiomyocytes were dissociated and plated on glass coverslips in culture medium before use. For action potential measurements, cells were placed in a small-volume chamber and bathed in Tyrode’s solution containing (mM) 140 NaCl, 5.4 KCl, 1.8 CaCl_2_, 1 MgCl_2_, 10 HEPES, 10 glucose, pH 7.4, maintained at 33–35 °C. Action potentials were recorded using a patch clamp amplifer (Axopatch 200B, Molecular Devices) in whole-cell current-clamp mode. Pipettes (1–3 MOhm) were filled with internal solution containing (mM) 100 K-aspartate, 30 KCl, 10 HEPES, 1 MgCl_2_, 5 Mg-ATP, 5 Na_2_ creatine phosphate, 0.1 EGTA, 0.025 CaCl_2_ (free Ca^2+^ 50 nM), pH 7.2. Intracellular Ca^2+^-dependent fluorescence arising from the GCaMP6s indicator was monitored simultaneously with action potential measurements using epi-illumination and a photomultiplier tube (D104, PTI). Recorded fluorescence was corrected for background and normalized to the baseline value (∆*F/F*_0_). In the case of spontaneously active cells, the baseline was estimated by extrapolating the fit of an exponential + constant function to the declining phase of the fluorescence transient.

### Myocardial infarction and cardiomyocyte injection

Myocardial infarction was performed by ligation of the left anterior descending coronary artery in NSG mice (Jackson Laboratory) under anesthesia with 1–3% isoflurane. Rhesus iPSCs-derived cardiomyocytes (5 × 10^5^/20 ul) were injected into the myocardium at the border zone of the infarct area with a 29-gauge needle 5 min after permanent LAD ligation. 2 months post surgery, the animals were euthanized and the hearts were removed and perfused with 4% paraformaldehyde for immunostaining. All mouse protocols were reviewed and approved by National Heart, Lung, and Blood Institute animal care and use committee.

### Gene targeting, colony selection and screening

On day 0, Lipofectamine 3000 transfection was performed as described in manufacturer’s protocol (Thermo Fisher Scientific) after RhiPSCs reach 30~40% confluency in 6-well plate. GECI donor and rhesus AAVS1-CRISPR/Cas9 plasmids were added (5 ug per well) in MEF-CM culture medium. Transfected RhiPSCs were passaged onto puromycin-resistant DR4 mouse embryonic fibroblast (MEF) feeders (GlobalStem) on day 2, and 0.5 ug/ml of puromycin (Sigma-Aldrich) was added on day 3, and continued for 10 days thereafter, for selection of targeted colonies. To screen for targeted and random integrations, Southern blot analysis was performed as previously described by Lofstrand Lab in Gaithersburg, Maryland. An AAVS1 5′ homology arm probe was used for Southern blot to recognize a wild type (WT) band at 6.7 kb and a targeted insertion (TI) band at 3.6 kb.

### Immunofluorescence staining

Immunofluorescence staining of iPSC colonies, differentiating progenitor cells, and RhiPSC-dervied cardiomyocytes were performed as previously described^10^. The following primary antibodies were used for immunofluorescence: NANOG (goat, 1:100, AF1997, R&D Systems); SSEA4 (mouse, 1:100, sc-21704, Santa Cruz); OCT3/4 (mouse, 1:100, sc-5279, Santa Cruz,); TRA-1-60 (mouse, 1:100, sc-21705, Santa Cruz); Sendai virus pAb (rabbit, 1:100, PD029, MBL); Brachyury (goat, 1:100, AF2085, R&D Systems); Tuj1 (mouse, 1:100, β- III Tubulin, MAB1195, R&D system); α-ACTININ (mouse, 1:1000, A7811, Sigma); cTnI (cardiac troponin I, rabbit, 1:200, sc-15368, Santa Cruz); cTnT (cardiac troponin T, mouse, CT3, 1:1000, DSHB); MYH (mouse, MF20,1:1000, DSHB); NKX2.5 (rabbit, 1:200, ab35842, Abcam); MLC2A (MYL7, mouse, 1:100, 311011, Synaptic System); MLC2V (MYL2, rabbit, 1:100, 10906-1-AP, ProteinTech).

### Karyotyping and *in vivo* teratoma formation

Standard G-banded karyotypes were obtained and interpreted by the Oregon Health and Science University (OHSU) research cytogenetics core laboratory. For teratoma formation, RhiPSCs were dissociated by TrypLE and injected subcutaneously (1 × 10^6^ cells/injection) into the hindlimbs of 6-week-old male NSG mice. Teratoma growth was assessed weekly and the tumors were collected after 6 weeks for paraffin embedding, sectioning and histological staining.

### Vector construction

G-CaMP3, pGP-CMV-GCaMP6s, CMV-G-GECO1.0, and CMV-NLS-R-GECO1.0 were purchased from Addgene (plasmid 22692, 40753, 32447, and 32462, respectively). Rhesus AAVS1-CRISPR/Cas9 and rhesus AAVS1-CAG-copGFP vector were described previously. The copGFP marker gene in rhesus AAVS1-CAG-copGFP vector was replaced with G-CaMP3, GCaMP6s, G-GECO1.0, or NLS-R-GECO1.0 following digestion with BsrGI and MluI and Gibson Assembly cloning.

### Live imaging, quantification analysis and drug studies of GECI RhiPSC-CM

Live imaging was performed on a Zeiss Axio Observer A1 microscope. A heated platform was used to maintain temperature at 37 °C. The GFP filter was used to view G-CaMP3, GCaMP6s, and G-GECO1.0. Videos were obtained using the time series mode in ZEN software at a rate of 10 fps. Quantification of fluorescence intensity was conducted using ZEN software. For drug testing, isoproterenol (10 uM) and verapamil (0.2 uM) were dissolved in DMSO. 0.1% DMSO was used as a control. Recordings were performed 10 minutes after treatment of isoproterenol and 3 minutes after treatment of verapamil.

### Statistical analysis

All data are shown as mean ± SEM of 3 or more independent experiments. Student’s *t* tests were utilized and a *p* value ≤ 0.05 was considered statistically significant.

## Electronic supplementary material

Supplementary methods and figures

Beating rhesus iPSC-cardiomyocytes

Beating rhesus iPSC-cardiomyocytes expressing GCaMP6
