# Supplementary material for: Efficient differentiation of cardiomyocytes and generation of calcium-sensor reporter lines from nonhuman primate iPSCs
Source: Sci Rep. 2018 Apr 12;8:5907. doi: 10.1038/s41598-018-24074-y (PMC5897327; doi:10.1038/s41598-018-24074-y)

Supplementary Data for

Efficient differentiation of cardiomyocytes and generation of calcium-sensor reporter lines from nonhuman primate iPSCs

Yongshun Lin[1*], Huimin Liu[1,2,5*], Michael Klein[3], John Ostrominski[4], So Gun Hong[4], Ravi Chandra Yada[4], Guibin Chen[2], Keron Navarengom[2], Robin Schwartzbeck[2], Hong San[6], Zu-Xi Yu[7], Chengyu Liu[8], Kaari Linask[1], Jeanette Beers[1], Lugui Qiu[5], Cynthia E. Dunbar[4], Manfred Boehm[2], and Jizhong Zou[1]

1. iPSC Core, National Heart, Lung, and Blood Institute (NHLBI), National Institutes of Health (NIH), Bethesda, Maryland, USA
2. Laboratory of Cardiovascular Regenerative Medicine, Center for Molecular Medicine, NHLBI, NIH, Bethesda, Maryland, USA
3. Uniformed Services University of Health Sciences, Bethesda, Maryland, USA
4. Hematology Branch, NHLBI, NIH, Bethesda, Maryland, USA
5. State Key Laboratory of Experimental Hematology, Institute of Hematology and Blood Diseases Hospital, Chinese Academy of Medical Science and Peking Union Medical College, Tianjin, China
6. Genome Techology Branch, National Human Genome Research Institute, NIH, Bethesda, Maryland, USA
7. Pathology Core, NHLBI, NIH, Bethesda, Maryland, USA
8. Transgenic Core, NHLBI, NIH, Bethesda, Maryland, USA

* These authors contribute equally

Supplementary methods

Real-time RT-PCR

RNA of the cells at different iPSC-CM differentiation stages was purified using TRI Reagent® Solution according to Ambion's protocol. Residual DNA was removed using the TURBO DNA-free™ kit. To generate cDNA, the reverse transcription (RT) was carried out with Maxima H Minus Reverse Transcriptase (Thermo Scientific) primed with Poly N15-mer (Eurofins) following the recommended protocol. Prior to PCR, RNA template was removed from cDNA with addition of Ambion Ribonuclease H. Real-time RT-PCR was performed on the BIO-RAD CFX96 using SsoAdvanced™ Universal SYBR® Green Supermix. PCR primers are listed in the supplementary table.

Flow cytometry

After 10 days of monolayer differentiation, cells were dissociated with TrypLE (Invitrogen) for 5-8 minutes. Single cells were washed off and suspended in culture media followed by centrifugation. The pellets were washed with PBS once, then fixed and permeabilized using 2% PFA / 0.1% Triton X-100 for 10 minutes. The cells were centrifuged and re-suspended with 1% BSA to block nonspecific binding of antibody, followed by incubation with primary antibodies (diluted in 1% BSA) for 1 hour. After washing, the cells were incubated with secondary antibodies conjugated with FITC (Invitrogen, 1:1000 diluted in PBS) for 30-60 minutes. The cells were washed once then suspended in PBS for the flow cytometry assay.

Supplementary figure legends

Supplementary Figure 1 Cardiac differentiation of RhiPSCs under CHIR-driven induction. **(a)** Phase contrast (left panel) and immunofluorescence staining (right panel) of neural cells (green) and cardiomyocytes (red) under the CHIR-driven cardiac differentiation conditions. Tuj1, class III β-tubulin; cTnI, cardiac troponin I. Nuclei were stained by DAPI (blue). Scale bars, 50µm. **(b)** Flow cytometry results with cardiac troponin T (cTnT) antibody showed the comparison of cardiac differentiation efficiency under different induction conditions (data shown are mean± SEM, n=3). CHIR, CHIR99021, ctrl, control; IP, IWP2.

Supplementary Figure 2 Effects of FGF2 treatment on loss of pluripotency and gaining of early mesoderm

identity. Real time PCR results showed the dynamic expression profile of pluripotecy markers (NANOG and POU5F1) and early mesoderm markers (T, MESP1, PDGFRa, and AXIN2) in the first four days after different combinations of growth factor treatments (data shown are mean± SEM, n=3). B, BMP4; A, Activin A; F, FGF2. The concentrations of BMP4 and Activin A were 10ng/ml; the concentrations (ng/ml) of FGF2 were listed.

Supplementary Figure 3 Representative images of morphology change during RhiPSC cardiac differentiation under different conditions from more than 3 independent experiments. **(a)** Phase contrast images of monolayer cell morphology at day 7 after different mesoderm induction conditions. CHIR, CHIR99021 (5μM); B, BMP4 (10ng/ml); A, Activin A (10ng/ml); F, FGF2. The concentrations (ng/ml) of FGF2 were listed. Scale bars, 200μm. **(b)** Phase contrast images of chronological morphology changes from day 1 to day 7 after different mesoderm induction conditions. CHIR, CHIR99021 (5uM); B, BMP4 (10ng/ml); A, Activin A (10ng/ml); F, FGF2 (10ng/ml). Scale bars, 100μm.

Supplementary Figure 4 Maintenance and cardiac differentiation of RhiPSCs on different matrix-coated plates. **(a)** Representative phase contrast images of RhiPSC colonies cultured on different matrices (Matrigel, Synthemax, and VTN-N) in MEF conditioned media. Scale bars, 100μm. **(b)** Representative phase contrast images of different cardiac differentiation stages of RhiPSCs on different matrices (Matrigel, Synthemax, and VTN, N). Scale bars, 100μm.

Supplementary Figure 5 Full length Southern blot showing clones from all four GECI RhiPSC lines.

Supplementary Figure 6 The comparison of CHIR and BAF regulation on *POU5F1*, *T*, and *MESP1* RNA expression during rhesus and human iPSC differentiation. The qPCR data are shown as mean± SEM (n=3)**..** Rhesus, CHIR, rhesus iPSCs under CHIR treatmentt Rhesus, BAF, rhesus iPSCs under BAF treatmentt human, CHIR, human iPSCs under CHIR treatment.

Supplementary Figure 1.
Cardiac differentiation of RhiPSCs under CHIR-driven induction

a

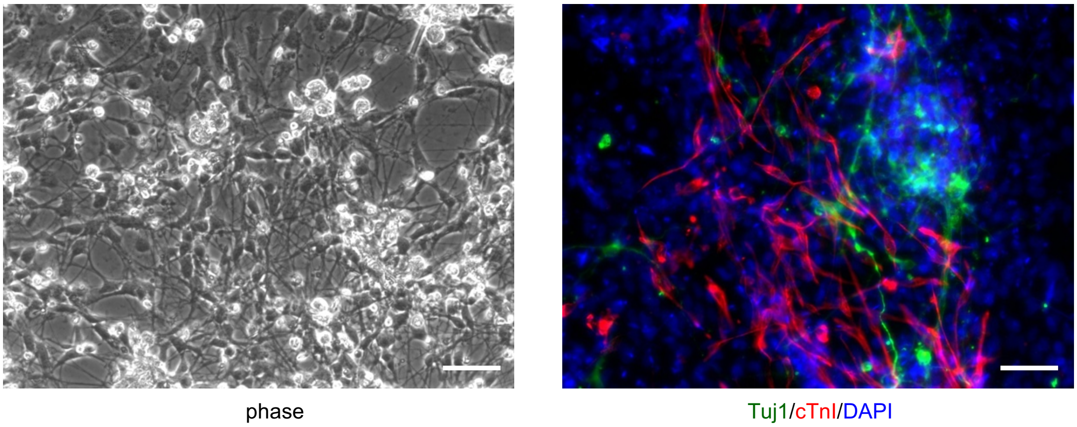

b

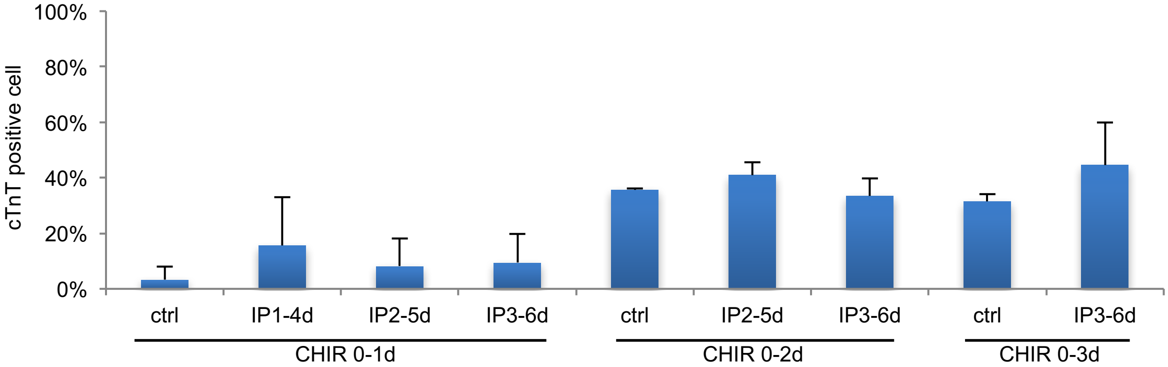

Supplementary Figure 2.
Effects of FGF2 treatment on loss of pluripotency and gaining of early mesoderm identity

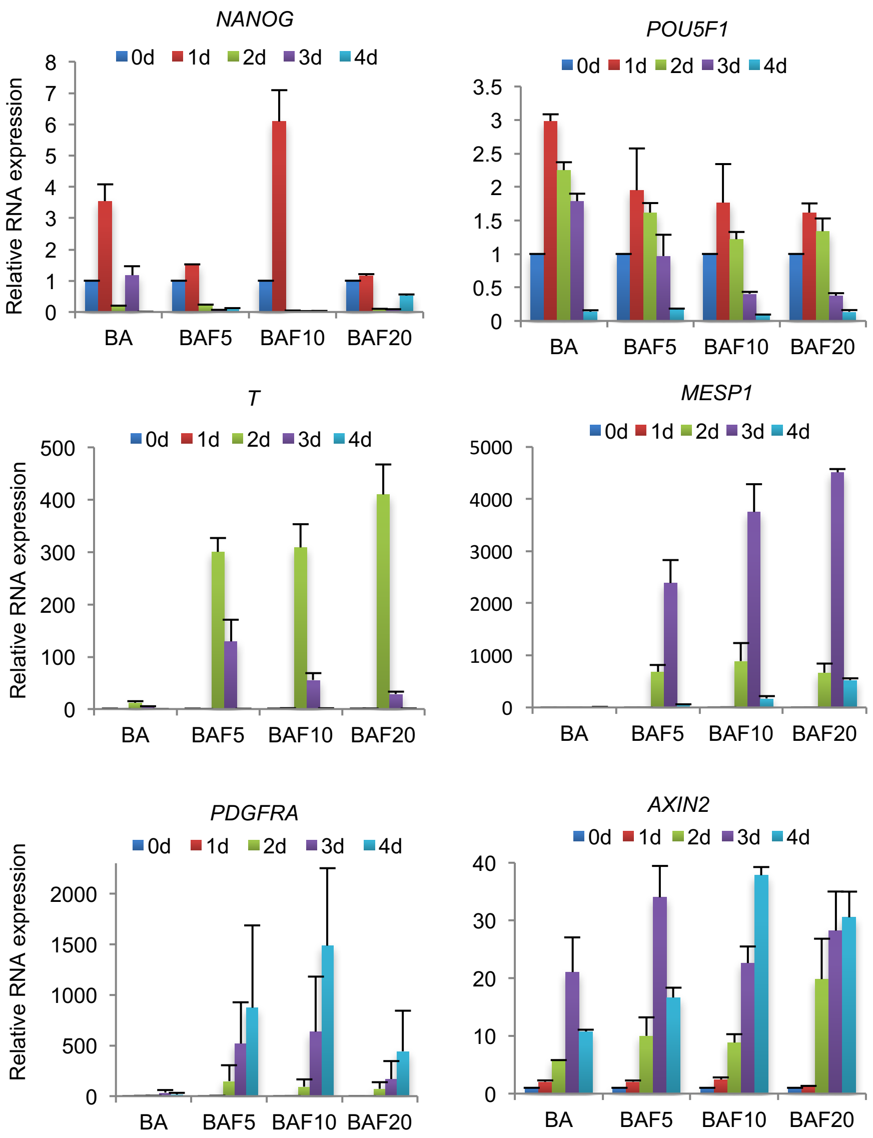

Supplementary Figure 3.
Chronological morphology change during RhiPSC cardiac differentiation under different conditions

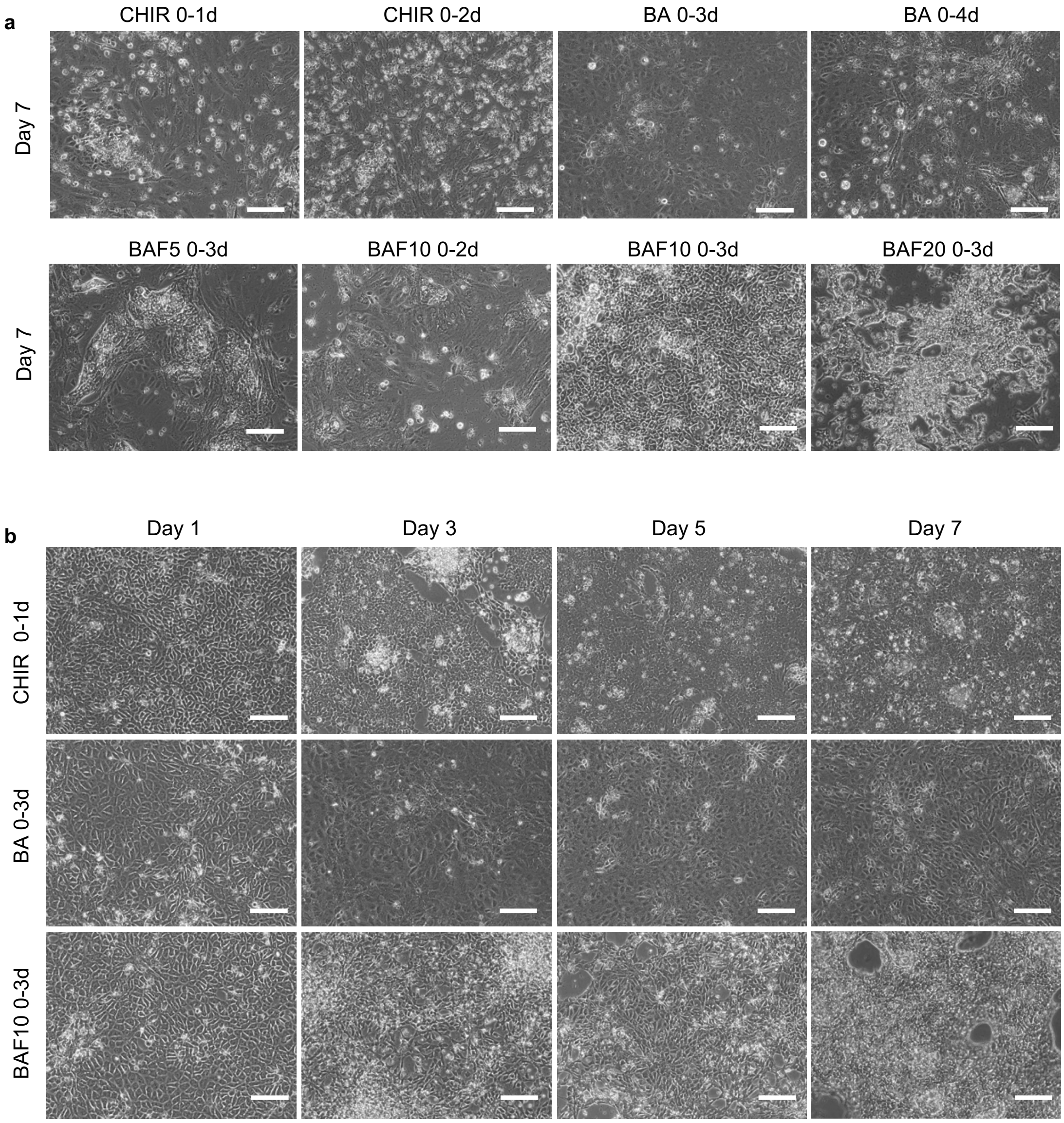

Supplementary Figure 4.
Maintenance and cardiac differentiation of RhiPSCs on plates coated with different matrices

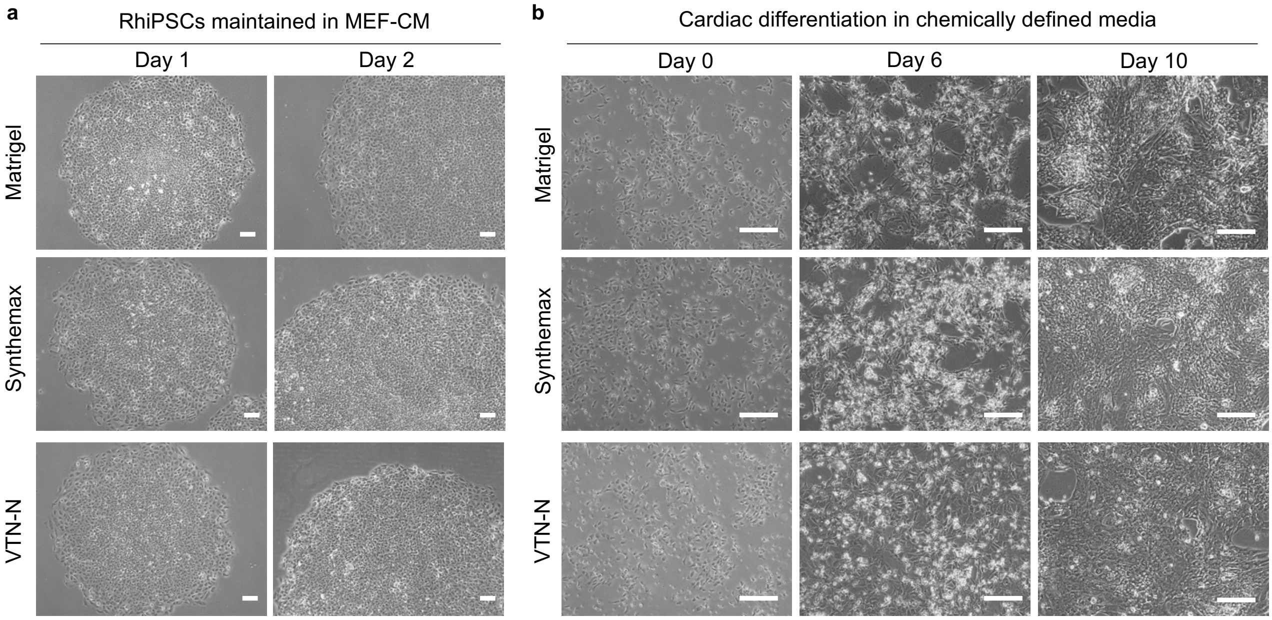

Supplementary Figure 5.
Full-length Southern blot of GECI RhiPSC lines

GCaMP3 GCaMP6s R-GECO1.0 G-GECO1.0

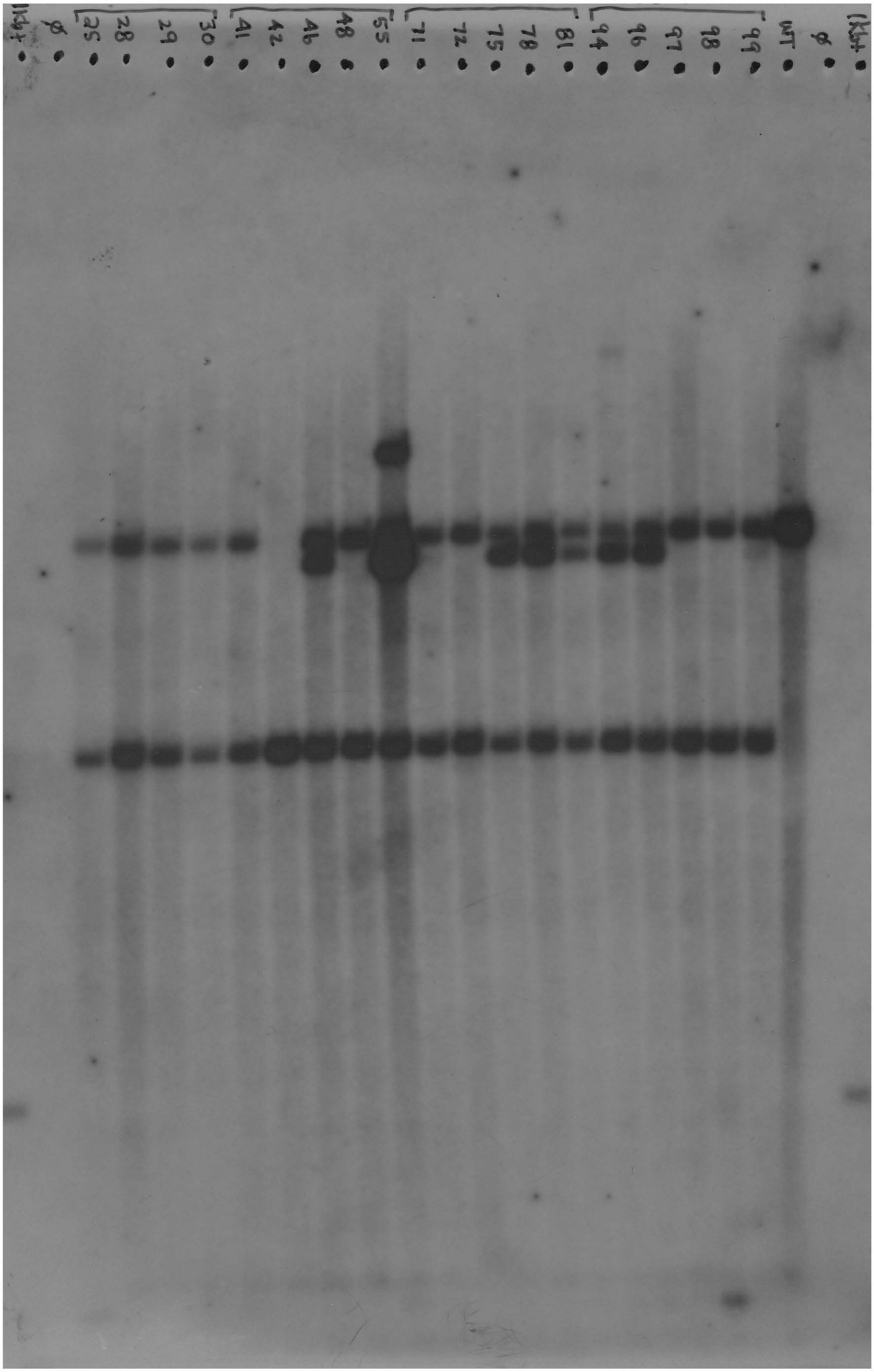

Supplementary Figure 6.
The comparison of CHIR and BAF regulation on POU5F1, T, and MESP1 expression during rhesus and human iPSC differentiation

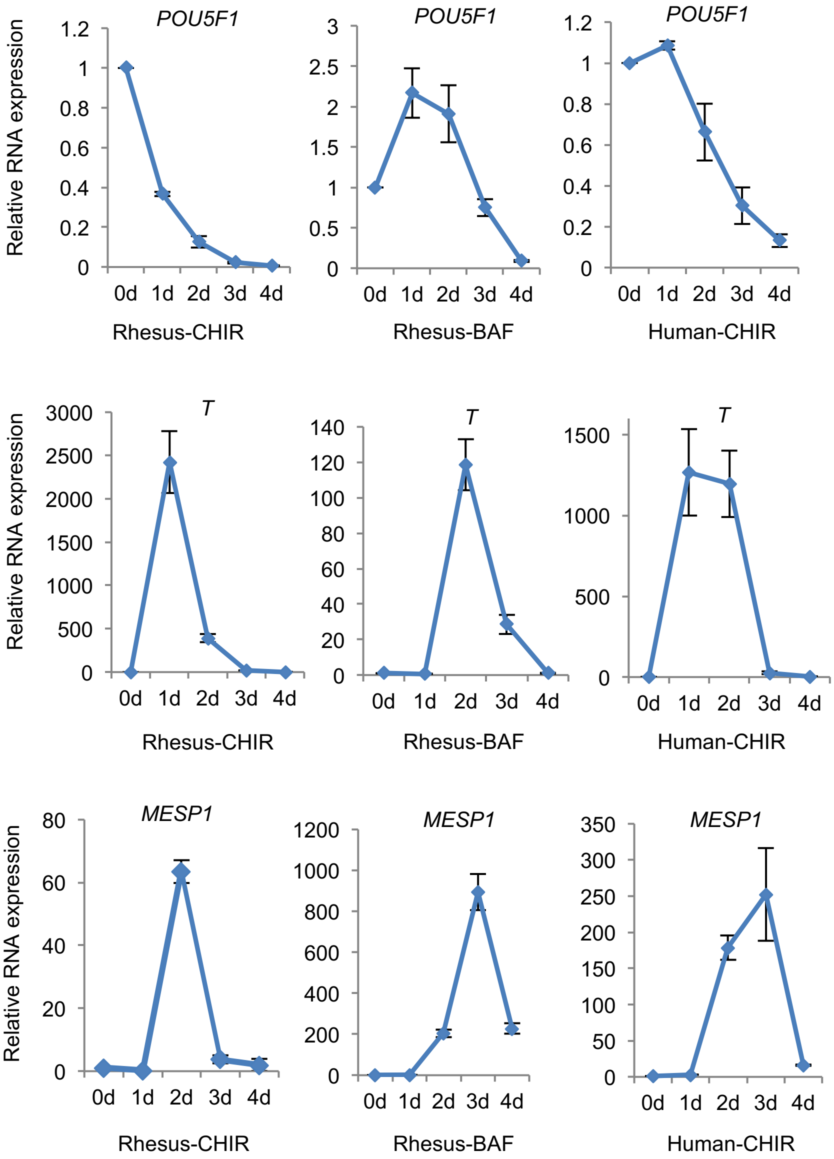

Supplement: Supplementary file 1 — Supplementary methods and figures [file 41598_2018_24074_MOESM1_ESM.pdf]
